# An increased incidence of avascular necrosis as the predisposing aetiology for primary total hip arthroplasty in sub-Saharan Africa – a retrospective review of 1,400 consecutive patients

**DOI:** 10.1051/sicotj/2025052

**Published:** 2025-09-24

**Authors:** Dyllan B. Geldenhuys, Josip Nenad Cakic, Lipalo Mokete, Nkhodiseni Sikhauli, Jurek Rafal Tomasz Pietrzak

**Affiliations:** 1 University of the Witwatersrand, Arthroplasty Unit, Department of Orthopaedic Surgery, Charlotte Maxeke Johannesburg Academic Hospital 1 Jubilee Road Parktown 2050 Johannesburg South Africa; 2 Life Fourways Hospital Fourways 2055 Johannesburg South Africa; 3 Busamed Modderfontein Hospital 2090 Johannesburg South Africa

**Keywords:** Total hip arthroplasty (THA), Avascular necrosis (AVN), South African Hospital, Human immunodeficiency virus (HIV), Osteoarthritis (OA)

## Abstract

*Introduction*: Worldwide, more than 1 million Total Hip Arthroplasties (THAs) are performed annually, with this number predicted to increase by 37.7% by the year 2060. This places a significant financial burden on the healthcare system, with the average cost of a THA being approximately $40,000. Several factors ultimately contribute to patient outcomes and complications, including surgical approach, surgeon’s experience, patient age, BMI, and most importantly, the preoperative diagnosis. Our paper aimed was to describe the various aetiologies of hip pathologies in patients presenting for primary elective THA to a tertiary academic sub-Saharan African institution. *Materials and methods*: We retrospectively reviewed 1400 consecutive patients presenting for elective primary THA between January 2015 and December 2021. Patients’ preoperative clinical notes, radiological records, and intraoperative results were independently assessed by two examiners to diagnose the hip pathology. A comparison of the presenting preoperative aetiologies was made between those seen in developed countries and those seen in more developing countries. *Results*: 2176 pathological hips were evaluated. Bilateral pathology was present in 56% of patients, of which 92% had the same pathology. There were 427 (31%) males and 973 (69%) females, with an average patient age of 58 ± 14.13 years and an average BMI of 31.01 ± 15.13 kg/m^2^. The preoperative aetiologies included primary osteoarthritis (OA) (*n* = 406 [29%]) and avascular necrosis of the femoral head (AVN) (*n* = 322 [23%]), of which (*n* = 162 (58%) had bilateral pathology. The primary cause of AVN was HIV (49%). Patients presenting with AVN were significantly younger (*p* < 0.0001) and had a lower BMI (*p* < 0.0001) in comparison to patients presenting for other pathologies. *Conclusion*: This study underscores the significance of aetiology in THA outcomes and highlights the unique challenges faced in developing countries. By identifying the specific causes of hip pathology in this population, healthcare providers can better allocate resources and develop tailored treatment strategies to improve outcomes in resource-limited settings.

## Introduction

Worldwide, more than one million total hip arthroplasties (THAs) are performed annually [1]. This figure is expected to rise significantly by 37.7% by the year 2060, driven in part by an aging global population, an increasing prevalence of hip pathologies, and improved access to surgical intervention [2]. Recent studies highlight a growing demand for THA in developed countries, attributed to advancements in surgical techniques and improvements in patient care [3]. Projections suggest that by 2060, the prevalence of THA in the United States of America (USA) and the United Kingdom (UK) will increase by 659% and 40%, respectively [2, 4].

Similarly, the demand for THA in developing countries is also increase, albeit at a slower pace [5]. This is attributed to several challenges, including limited healthcare resources, financial constraints, higher costs, and a shortage of trained orthopaedic surgeons [5]. Developing countries face unique hurdles that impact the availability and quality of THA services, leading to disparities in access and outcomes compared to developed nations [5]. Graham et al. conducted a review of Malawi’s national joint registry and reported that between 2005 and 2008, a total of 70 THAs were performed over the 3 years, underscoring the limited access to such procedures in the region [6]. Similarly, Lisenda et al. reported that between 2009 and 2015, barely 153 THAs were performed at one of Botswana’s major referral hospitals over a six-year period [7].

Despite these challenges, the outcomes of THA are generally favourable, with reported patient satisfaction rates exceeding 90% and implant survivorship of up to 95% at 10 years and 80% at 25 years [8]. Nonetheless, the ultimate success of THA is still influenced by various factors, including patient demographics, implant selection, surgical technique, and postoperative care [9]. Another critical factor is the preoperative aetiology of hip pathology, which not only influences outcomes but also plays a vital role in resource allocation and surgical planning, critical in resource-constrained settings like developing countries. Understanding the aetiology is thus crucial and helps to tailor treatment strategies, optimize resource utilization, and essentially improve overall patient outcomes [10].

While extensive research has characterized the distribution of hip pathologies in developed nations such as the United States and the United Kingdom, a crucial gap exists in understanding these patterns in developing countries, particularly in sub-Saharan Africa. South Africa presents a unique epidemiological landscape with a confluence of factors not typically seen in developed nations, including a high burden of HIV infection, limited access to early healthcare intervention, and diverse socioeconomic determinants of health. Therefore, the study aimed to elucidate the aetiology of hip pathology in patients presenting for THA in South Africa.

## Materials and methods

We performed a retrospective analysis of the hip pathology of 1,400 consecutive patients who presented to a tertiary, academic referral centre in South Africa for THA between January 2015 and December 2021. This specialist institution serves patients from four surrounding South African provinces and neighbouring Southern African Development Community (SADC) countries, including Zimbabwe, Botswana, Mozambique, Zambia, Lesotho, and as far north as the Democratic Republic of Congo and even Nigeria.

Preoperatively, all patients underwent a routine, institution-specific diagnostic workup that included clinical, radiological, and serological investigations. All patients were assessed using the same clinical history and examination template, delineating previous medical history, previous surgical history, family history, and evaluation of clinical notes from other specialty clinics at the same institution, including Rheumatology, Pulmonology, Cardiology, and the Infectious Diseases units, amongst others. All patients underwent standard radiological evaluation, which included a standing pelvic anteroposterior (AP), false-profile hip views, and lateral hip X-rays. Routine serological tests for all patients included a Full Blood Count (FBC), Urea and Creatinine, Albumen level, and screening for Human Immunodeficiency Virus (HIV). In the event of patients testing positive for HIV, a CD4+ count and viral load (VL) were performed.

Patients presenting for revision THA, THA for femoral neck fractures (FNFs), and those with incomplete perioperative diagnostic work-up were excluded from this study. Patients who underwent THA and those who underwent staged bilateral THA due to the same diagnosis were included as one case.

All clinical notes, results of serological investigations, and X-rays were independently assessed by two examiners to diagnose the aetiology of the hip pathology. In cases of disagreement, the senior author resolved the deadlock.

Ultimately, all patients were divided into six groups based on the aetiology of their hip pathology for THA: avascular necrosis of the femoral head (AVN), primary osteoarthritis (OA), adult hip dysplasia (ADH), femoroacetabular impingement (FAI), paediatric disorders (including developmental dysplasia of the hip [DDH], slipped capital femoral epiphysis [SCFE], and Perthes disease), and secondary osteoarthritis (OA), which resulted from prior trauma, malignancy, infectious processes, or inflammatory arthropathies (namely, rheumatoid arthritis, ankylosing spondolytis, and psoriatic arthritis).

Subsequently, once the diagnosis was made, the patients were categorised according to the pre-operative aetiology, with the severity of the presenting pathology being assessed using an appropriate and related classification system.

## Results

In total, we reviewed 1,400 consecutive patients who presented for elective primary THA to our institution between January 2015 and December 2021. This included 2,176 pathological hips, of which 776 (56%) patients had bilateral hip pathology and 624 (44%) had unilateral hip pathology.

There were 427 (31%) males and 973 (69%) females. The mean patient age across the entire group was 58 ± 14.13 years, with an average BMI of 28.01 ± 5.13 kg/m^2^. Of all examined patients, 322 (23%) were overweight (BMI: 25–29 kg/m^2^), 644 (46%) were obese (BMI: 30–49 kg/m^2^), and 336 (24%) were super obese (BMI: >50 kg/m^2^). There were 425 (30.3%) with hypertension, 332 (23.7%) with diabetes mellitus, and 210 (15.1%) with a history of previous trauma. The HIV seroprevalence in this cohort was 15.8% (*n* = 228). See Table 1 for further demographic details.

**Table 1:** Demographic data of the total number of patients.

	*n* (%)
Total number of patients	1400
Total number of hips	2176
Left	979 (45%)
Right	1197 (55%)
Unilateral	624 (44%)
Bilateral	776 (56%)
Gender	
Male	427 (31%)
Female	973 (69%)
Age (years) (±SD)	58.80 ± 14.13
BMI (kg/m^2^) (±SD)	31.01 ± 15.13
ASA	
1	223 (16%)
2	911 (65%)
3	266 (19%)
Number of co-morbidities	
0	223 (16%)
1	381 (27%)
2	545 (39%)
≥3	251 (18%)
Co-morbidities/risk factors	
HIV	228 (15.8%)
Previous trauma	210 (15.1%)
Rheumatoid arthritis	120 (8.5%)
Tuberculosis	56 (4%)
Alcohol use	155 (11%)
COPD	40 (2.8%)
SLE	30 (2.2%)
Sickle cell disease	16 (1.1%)
Ischemic heart disease	25 (1.7%)
Malignancy	35 (2.5%)
Diabetes	332 (23.7%)
Hypertension	425 (30.3%)
Hypothyroidism	69 (4.9%)

### Overall

**Figure 1:**
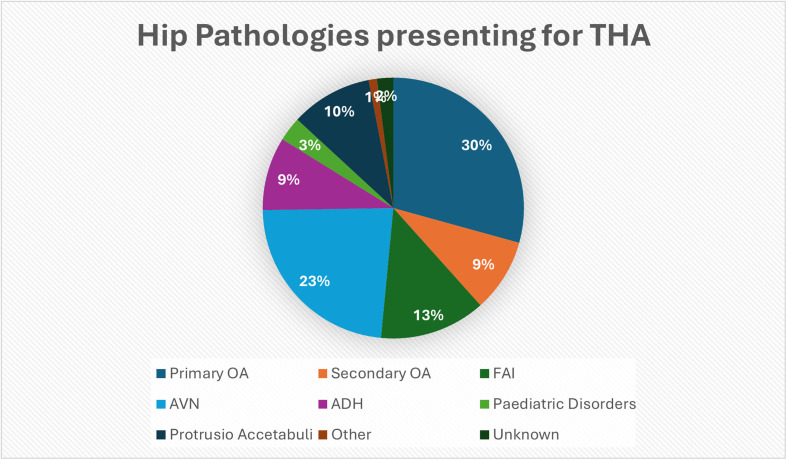
Breakdown of various pathologies in patients presenting for THA.

In total, there were 406 (29%) patients with primary OA and 126 (9%) with secondary OA. Additionally, 322 (23%) patients were diagnosed with AVN, 182 (13%) patients with FAI, and 42 (3%) patients presented with paediatric disorders. See Figure 1 for a breakdown of hip pathologies.

Among patients with a preoperative diagnosis of OA, the mean age at presentation was 71.8 years, while those with secondary OA, AVN, FAI, and ADH presented at mean ages of 60.3, 52.4, 55, and 61.3 years, respectively (*p* < 0.0001). The average BMI in the OA group was 34.3 kg/m^2^, compared to 26.5 and 26.8 kg/m^2^ in the secondary OA and AVN cohorts (*p* = 0.001). In terms of laterality, 187 (46%) patients with OA had unilateral hip pathology and 219 (54%) had bilateral involvement. For patients with AVN and FAI, bilateral hip pathology was observed in 187 (58%) and 100 (55%) cases (*p* = 0.001). Across the various aetiologies, there were 231 (55%) patients with hypertension and 186 (52%) patients with diabetes mellitus in the OA group. In the secondary OA group, 94 (75%) patients had rheumatoid arthritis, while in the group of patients with AVN, 158 (49%) patients were HIV positive. See Table 2 for further details pertaining to the various hip pathologies.

**Table 2:** Breakdown of the details of all potential hip pathologies.

*n* = Number of patients	Primary OA	Secondary OA	FAI	AVN	ADH	Paeds	Protrusio accetabuli	Other	Unknown	*P*-value
	(*n* = 406)	(*n* = 126)	(*n* = 182)	(*n* = 322)	(*n* = 126)	(*n* = 42)	(*n* = 140)	(*n* = 42)	(*n* = 14)	
Age (years) (±SD)	71.8 ± 13.7	60.3 ± 16.1	55 ± 12.59	52.4 ± 13.08	61.3 ± 10.75	53 ± 14.21	57 ± 23.03	56 ± 11.56	61 ± 12.48	<0.0001
Gender (*n*; %)										
Male	122 (30%)	53 (42%)	64 (35%)	122 (38%)	40 (32%)	14 (33%)	28 (20%)	12 (29%)	6 (47%)	0.001
Female	284 (70%)	73 (48%)	118 (65%)	200 (42%)	86 (68%)	28 (67%)	112 (80%)	30 (71%)	8 (53%)	
BMI (kg/m^2^) (±SD)	34.3 ± 6.69	26.5 ± 4.12	28 ± 3.74	26.8 ± 5.17	31.2 ± 6.93	29 ± 7.24	28.3 ± 7.61	32 ± 4.36	30.6 ± 6.71	<0.0001
Unilateral	187 (46%)	16 (13%)	82 (45%)	135 (42%)	53 (42%)	22 (53%)	13 (9%)	19 (46%)	8 (56%)	0.001
Bilateral (*n*; %)	219 (54%)	110 (87%)	100 (55%)	187 (58%)	73 (48%)	20 (47%)	127 (91%)	23 (54%)	6 (44%)	
Co-morbidities (*n*; %)										
HIV positive	10 (2%)	7 (6%)	8 (5%)	158 (49%)	5 (4%)	3 (7%)	49 (38%)	2 (7%)	2 (6%)	
RA	4 (1%)	94 (75%)	2 (1%)	3 (1%)	3 (2%)	1 (2%)	10 (8%)	1 (4%)	2 (6%)	
TB	12 (2.8%)	3 (2%)	1 (0.5%)	29 (10%)	0 (0%)	2 (5%)	3 (2%)	6 (21%)	0 (0%)	
COPD	4 (1%)	3 (2%)	2 (1%)	26 (10%)	1 (0.7%)	0 (0%)	2 (1.5%)	1 (1%)	0 (0%)	
Malignancy	11 (2.6%)	5 (4%)	5 (2.9%)	3 (1%)	2 (1.5%)	1 (2%)	4 (3%)	3 (4%)	1 (3%)	
IHD	10 (7%)	3 (2.4%)	2 (1.1%)	4 (1%)	1 (0.7%)	0 (0%)	1 (0.8%)	2 (3%)	3 (9%)	
Diabetes	186 (52%)	22 (17%)	15 (8.8%)	42 (15%)	28 (21%)	8 (18%)	17 (13%)	10 (13%)	4 (13%)	
Hypertension	231 (55%)	41 (33%)	26 (15%)	71 (25%)	15 (11%)	8 (18%)	21 (17%)	5 (7%)	7 (22%)	
Hypothyroidism	18 (4.3%)	12 (9.6%)	4 (2.3%)	14 (5%)	6 (4.5%)	1 (2%)	10 (8%)	3 (4%)	1 (3%)	

BMI, Body mass index; HIV, Human Immunodeficiency Virus; RA, Rheumatoid Arthritis; TB, Tuberculosis; COPD, Chronic obstructive pulmonary disease; IHD, ischemic heart disease.

### Grading the various aetiologies (number of hips)

The various aetiologies of hip pathology with their relevant classification and grading system are presented in Tables 3 and 4.

**Table 3:** Total number of hips with sub-classifications.

Total number of hips (*n*; %)	2176
Primary osteoarthritis (OA)	638 (29%)
Tönnis 1	13 (2%)
Tönnis 2	236 (37%)
Tönnis 3	389 (61%)
Secondary osteoarthritis	198 (9%)
Post traumatic	31 (16%)
Post infective	10 (5%)
Inflammatory	149 (75%)
Malignancy	8 (4%)
Femoroacetabular impingement (FAI)	289 (13%)
Cam	109 (37.7%)
Pincer	51 (17.6%)
Combined	129 (44.7%)
Avascular Necrosis (AVN)	526 (23%)
Ficat & Arlet 1	0 (0%)
Ficat & Arlet 2	57 (11%)
Ficat & Arlet 3	190 (36%)
Ficat & Arlet 4	279 (53%)
Adult dysplastic hip (ADH)	186 (9%)
Hartofilakidis A	78 (42%)
Hartofilakidis B	97 (52%)
Hartofilakidis C	11 (6%)
Crowe 1	65 (35%)
Crowe 2	107 (58%)
Crowe 3	13 (7%)
Paediatric disorders	56 (3%)
Perthes	36 (64%)
DDH	15 (27%)
SCFE	5 (9%)
Protrusio accetabuli	223 (10%)
Other	28 (1%)
Unknown	32 (2%)

**Table 4:** Patients with AVN who underwent THA.

	Total number of patients	Alcohol	HIV	Trauma	Corticosteroid use	Sickle cell disease	Other	*P*-value
	*n* = 322 (%)	*n* = 58 (18%)	*n* = 158 (49%)	*n* = 80 (25%)	*n* = 19 (6%)	*n* = 3 (1%)	*n* = 4 (1%)	
Age (Years) (±SD)								
Gender (*n*; %)	52.4 ± 13.08	54.35 ± 10.2	51.4 ± 14.04	54.1 ± 10.94	52.2 ± 2.82	48.1 ± 15.11	51.2 ± 9.51	0.5742
Male	122 (38%)	28 (48%)	88 (56%)	46 (57%)	7 (39%)	1 (34%)	3 (75%)	0.676
Female	200 (62%)	30 (52%)	70 (44%)	34 (43%)	12 (61%)	2 (66%)	1 (33%)	
BMI (kg/m^2^) (±SD)	28 ± 3.74	28.5 ± 5.46	26.8 ± 9.89	26.45 ± 3.01	26.5 ± 5.86	28.1 ± 4.96	25.1 ± 8.16	0.6774
Unilateral	135 (42%)	34 (58%)	46 (29%)	68 (85%)	8 (44%)	2 (66%)	3 (75%)	<0.0001
Bilateral (*n*; %)	187 (58%)	24 (42%)	112 (71%)	12 (15%)	11 (56%)	1 (34%)	1 (25%)	
Ficat & Arlet grade (Number of hips) (*n*; %)	526 (100%)	95 (18%)	257 (49%)	132 (25%)	32 (6%)	5 (1%)	6 (1%)	
1	0 (0%)	0 (0%)	8 (3%)	7 (5%)	1 (2%)	0 (4%)	0 (3%)	
2	57 (11%)	5 (5%)	21 (8%)	20 (15%)	3 (10%)	1 (12%)	0 (4%)	
3	190 (36%)	37 (39%)	82 (32%)	55 (42%)	11 (33%)	2 (41%)	3 (56%)	
4	279 (53%)	53 (56%)	146 (57%)	50 (38%)	17 (55%)	2 (43%)	3 (37%)	
Co-morbidities (*n*; %)								
RA	3 (1%)	1 (2%)	14 (9%)	0 (0%)	0 (0%)	0 (0%)	1 (1%)	
TB	32 (10%)	0 (0%)	22 (14%)	2 (3%)	4 (24%)	1 (33%)	1 (50%)	
COPD	32 (10%)	7 (12%)	11 (7%)	9 (11%)	1 (6%)	0 (0%)	0 (0%)	
Malignancy	3 (1%)	1 (2%)	2 (1%)	1 (1%)	0 (0%)	0 (0%)	0 (0%)	
IHD	3 (1%)	1 (2%)	2 (7%)	1 (1%)	1 (1%)	0 (0%)	0 (0%)	
Diabetes	48 (15%)	19 (33%)	74 (47%)	3 (4%)	1 (6%)	1 (33%)	1 (50%)	
Hypertension	80 (25%)	16 (27%)	85 (54%)	3 (4%)	2 (12%)	1 (33%)	1 (50%)	
Hypothyroidism	16 (5%)	3 (6%)	47 (30%)	1 (1%)	0 (0%)	0 (0%)	0 (0%)	

In the group of patients diagnosed with primary OA, 13 hips (2%) were classified as Tönnis grade 1, 236 hips (37%) as Tönnis grade 2, and 389 hips (61%) as Tönnis grade 3.

Of the patients diagnosed with secondary OA, 31 hips (16%) were due to previous trauma, 10 hips (5%) resulted from an infectious process, 149 hips (75%) were associated with inflammatory arthropathy, and 8 hips (4%) were in patients with a history of current or past malignancy.

Among patients with AVN, 158 (49%) were associated with HIV seropositivity, 58 (18%) were secondary to alcohol abuse, 80 (25%) resulted from prior hip trauma, 3 (1%) were linked to sickle cell disease, and 19 (6%) were associated with long-term corticosteroid use. No hips were classified as Ficat and Arlet stage 1; 57 hips (11%) were classified as stage 2, 190 hips (36%) as stage 3, and 279 hips (53%) as stage 4.

Within the hips with FAI, 109 (37.7%) were classified as having Cam morphology, 51 (17.6%) as Pincer morphology, and 129 (44.7%) as combined pathology.

In the cohort of patients with ADH, 78 hips (42%) were classified as Hartofilakidis A, 97 hips (52%) as Hartofilakidis B, and 11 hips (6%) as Hartofilakidis C. According to the Crowe classification, 65 hips (35%) were Crowe 1, 107 hips (58%) were Crowe 2, and 13 hips (7%) were Crowe 3.

In the subgroup of patients with paediatric disorders, there were 36 (64%) patients who presented with Perthes disease, while DDH was diagnosed in 15 (27%) patients, and 5 (9%) patients had a SCFE.

## Discussion

To the author’s knowledge, this study is the first of its kind to evaluate the aetiologies necessitating THA within a Sub-Saharan African population. While this study was performed in a developing country, the incidence of primary OA in this cohort was 29%, making it the most prevalent hip pathology requiring THA in this population. However, this figure is notably lower than that reported in previous studies examining preoperative aetiologies for THA [11–13] The Joint Registry (NJR) covers England, Wales, Northern Ireland, the Isle of Man, and Guernsey, and remains to be the largest orthopaedic registry in the world, with just under 4 million procedure records submitted [11]. The NJRs’ 2023 annual report, for instance, reported that primary OA accounted for 91% of THA cases [12]. Similarly, Martinkovich et al. evaluated the patient characteristics in 429 patients who underwent primary elective THA in the USA and noted that primary OA was the preoperative diagnosis in 95% of patients [13].

In this study, we found a significantly higher prevalence of AVN as the underlying cause for THA compared to the rates observed in developed countries [13]. AVN was identified as the aetiology for THA in 23% of patients, which is consistent with the prevalence reported in other developing countries [14]. Ma et al. examined the preoperative aetiology for THA in 7663 patients across 13 hospitals in China and noted that AVN and DDH were the leading diagnoses, accounting for 45% and 30% of cases, respectively, while primary OA was diagnosed in only 12% of patients [15]. Comparably, Yakkanti et al. conducted an analysis of the incidence of various hip pathologies among 245,349 patients who underwent THA in India and the USA between 2016 and 2017. They reported that the proportion of patients with a preoperative diagnosis of AVN was significantly higher in India than in the USA, with rates of 51.8% and 5.97%, respectively (*p* < 0.001). [14]. Both of the above papers highlight the substantial differences in hip pathology between developed and developing countries, likely reflecting disparities in healthcare access, cost, and underlying health conditions.

Our study revealed that nearly half of the patients diagnosed with AVN were HIV-positive. The incidence of AVN as a consequence of previous hip trauma and a history of alcohol abuse was reported in 25% and 18% of cases, respectively. Specifically, HIV was identified as the underlying cause of AVN in 49% of cases. This contrasts with the findings by Kumar et al. and Vardhan et al., who assessed common pathologies necessitating THA in India and reported that 50% of cases were attributed to AVN, with alcohol use and long-term corticosteroid intake as the predominant etiological factors in 20% and 37% of cases in their series, specifically [16, 17]. Similarly, Tan et al. reviewed 2945 hips in patients undergoing THA in China, noting that corticosteroids, alcohol use, and trauma accounted for 27%, 37% and 15% of AVN cases, respectively, in their population [18]. In contrast, Boontanapibul et al. retrospectively reviewed the causes of atraumatic osteonecrosis in 937 patients below the age of 65 who presented for THA in the USA. Their findings revealed that corticosteroid use accounted for 35% of cases, excessive alcohol intake contributed to 11%, and 6% was attributed to sickle cell disease and HIV [19]. These disparities underscore the necessity for region-specific strategies in managing hip pathology and planning THA procedures.

Subsequently, the above comparisons and differences may highlight the environmental influences on hip pathology. The prevalence of HIV in the general population in India and China has been reported as 0.21% and 1%, respectively [20]. In contrast, South Africa has an estimated 8.45 million people living with HIV, representing 19.6% of its population and a prevalence rate of 13.9%, placing it among the countries with the highest HIV burden globally [21]. This high prevalence likely contributes to the greater incidence of AVN observed in this population compared to other regions. It emphasizes how regional health factors, particularly HIV, influence the prevalence of AVN and THA outcomes. In fact, we previously showed in 2020 that the seroprevalence of patients presenting for TKA and THA to our institution was 6.9% and 14.9%, respectively [22]. This study further corroborates the findings of Maharaj et al., with our current data showing that 15.8% of patients undergoing THA at our institution are seropositive.

In addition to differences in HIV prevalence, other regional factors may account for the disparity in AVN rates. In South Africa, delayed access to antiretroviral therapy (ART), particularly in earlier years of the HIV epidemic, may have contributed to prolonged periods of immune dysregulation and associated complications, including AVN [23]. Furthermore, the widespread use of corticosteroids in the treatment of tuberculosis – a disease highly endemic in South Africa – may be a contributing factor, as steroid use is a well-established risk factor for AVN [24]. These contextual differences may explain the higher burden of HIV-associated AVN observed in South Africa compared to countries such as India and China, where ART rollout timelines, healthcare access, and TB management practices differ significantly.

Similarly, AVN is associated with poorer outcomes following THA compared to primary OA. Subsequent anatomical alterations as a result of compromised femoral vascularity lead to subchondral bone collapse and structural deficiencies, which exacerbate technical challenges. This, coupled with potential systemic comorbidities such as chronic corticosteroid use or immunosuppression, may adversely affect implant fixation and long-term survivorship. A systematic review of more than 2 million THAs from 14 observational studies by Salman et al. reported an almost two times increased incidence of both revision THA and PJI in THA performed for AVN as opposed to OA. Correspondingly, a statistically increased incidence of periprosthetic femur fracture in the AVN group also existed [25]. Furthermore, using the Nationwide Readmissions Database, Sax et al. highlighted that THA for AVN resulted in a longer length of hospital stay and induced both a higher 90-day readmission costs and was associated with a greater prevalence of complications within 90 days in patients with AVN than OA [26].

In South Africa, the incidence of AVN is notably higher than in many high-income countries, driven by a complex interplay of contributing factors, including high rates of alcohol abuse, HIV-associated vasculopathy, and tuberculosis-related steroid therapy. Recognizing AVN as a prevalent and distinct indication for THA in the South African setting is therefore critical, as it demands tailored surgical planning and may influence implant selection, rehabilitation protocols, and patient counselling. Moreover, an evolution in the use of healthcare resources towards early detection, prevention strategies, and patient education may occur.

A key limitation of this retrospective study is the reliance on existing records and imaging, which may introduce bias due to incomplete or inconsistent documentation. Data quality and completeness varied across patients, and post hoc X-ray analysis may have led to misclassification of disease severity or aetiology. Interpretation of radiographs can be subjective and influenced by the assessor’s experience.

Selection bias is also a concern, as the cohort may not reflect the general population. Patients undergoing THA typically have advanced disease, potentially skewing findings toward more severe pathology. Additionally, inclusion of bilateral hips may have affected outcomes, as disease progression in one hip could influence the assessment of the contralateral side.

## Conclusion

This study underscores the significance of aetiology in THA outcomes and highlights the unique challenges faced in developing countries. By identifying the specific causes of hip pathology in this population, healthcare providers more effectively allocate resources and develop targeted strategies to improve outcomes in resource-limited settings.

## Data Availability

The datasets used and analysed during the current study are available from the corresponding author on reasonable request.
